# Association of Lipoprotein (a) variants with risk of cardiovascular disease: a Mendelian randomization study

**DOI:** 10.1186/s12944-021-01482-0

**Published:** 2021-06-01

**Authors:** Juan Xia, Chunyue Guo, Kuo Liu, Yunyi Xie, Han Cao, Wenjuan Peng, Yanyan Sun, Xiaohui Liu, Bingxiao Li, Ling Zhang

**Affiliations:** Department of Epidemiology and Health Statistics, School of Public Health, Capital Medical University and Beijing Municipal Key Laboratory of Clinical Epidemiology, No 10 Xitoutiao, You’anmenwai, Fengtai, Beijing, 100069 P. R. China

**Keywords:** Lipoprotein (a), Cardiovascular risk, Atrial fibrillation, Arrhythmia, Congestive heart failure, Ischemic stroke, East Asian

## Abstract

**Background:**

There is a well-documented empirical relationship between lipoprotein (a) [Lp(a)] and cardiovascular disease (CVD); however, causal evidence, especially from the Chinese population, is lacking. Therefore, this study aims to estimate the causal association between variants in genes affecting Lp(a) concentrations and CVD in people of Han Chinese ethnicity.

**Methods:**

Two-sample Mendelian randomization analysis was used to assess the causal effect of Lp(a) concentrations on the risk of CVD. Summary statistics for Lp(a) variants were obtained from 1256 individuals in the Cohort Study on Chronic Disease of Communities Natural Population in Beijing, Tianjin and Hebei. Data on associations between single-nucleotide polymorphisms (SNPs) and CVD were obtained from recently published genome-wide association studies.

**Results:**

Thirteen SNPs associated with Lp(a) levels in the Han Chinese population were used as instrumental variables. Genetically elevated Lp(a) was inversely associated with the risk of atrial fibrillation [odds ratio (OR), 0.94; 95% confidence interval (95%CI), 0.901–0.987; *P* = 0.012)], the risk of arrhythmia (OR, 0.96; 95%CI, 0.941–0.990; *P* = 0.005), the left ventricular mass index (OR, 0.97; 95%CI, 0.949–1.000; *P* = 0.048), and the left ventricular internal dimension in diastole (OR, 0.97; 95%CI, 0.950–0.997; *P* = 0.028) according to the inverse-variance weighted method. No significant association was observed for congestive heart failure (OR, 0.99; 95% CI, 0.950–1.038; *P* = 0.766), ischemic stroke (OR, 1.01; 95%CI, 0.981–1.046; *P* = 0.422), and left ventricular internal dimension in systole (OR, 0.98; 95%CI, 0.960–1.009; *P* = 0.214).

**Conclusions:**

This study provided evidence that genetically elevated Lp(a) was inversely associated with atrial fibrillation, arrhythmia, the left ventricular mass index and the left ventricular internal dimension in diastole, but not with congestive heart failure, ischemic stroke, and the left ventricular internal dimension in systole in the Han Chinese population. Further research is needed to identify the mechanism underlying these results and determine whether genetically elevated Lp(a) increases the risk of coronary heart disease or other CVD subtypes.

**Supplementary Information:**

The online version contains supplementary material available at 10.1186/s12944-021-01482-0.

## Background

Cardiovascular disease (CVD) is a class of diseases that include coronary artery diseases, stroke, heart failure, hypertensive heart disease, rheumatic heart disease, cardiomyopathy, abnormal heart rhythms, congenital heart disease, valvular heart disease, carditis, aortic aneurysms, peripheral artery disease, thromboembolic disease, and venous thrombosis [1]. CVD is the leading cause of death in China and worldwide [2, 3], accounting for two out of every five deaths in China, according to the China Cardiovascular Disease Report 2019 [4]. It has been estimated that the number of patients with CVD in China is over 330 million, and the prevalence of CVD continues to rise [4]. Recently, a study by Ooi EM and colleagues showed that the concentration of lipoprotein (a) [Lp(a)] is independently associated with angiographic extent and severity of coronary artery disease [5], a study by Rallidis LS and colleagues showed that high levels of Lp(a) are continuously and independently associated with an increased risk of acute coronary syndrome [6]; and a study by Verbeek R and colleagues showed that Lp(a) and low-density lipoprotein cholesterol are independently associated with CVD risk [7]. There is a well-documented empirical relationship between Lp(a) and CVD [8]; however, causal evidence, especially from the Chinese population, is lacking.

Confounding or reverse causation are two major problems in observational studies that render conclusions about causality uncertain. The Mendelian randomization (MR) approach has been developed to assess whether an association represents a causality in epidemiologic settings [9]. For example, the results of an observational study showed that Lp(a) is an independent risk factor associated with the progression of intima-media thickness [10], while the results from MR did not support an effect of Lp(a) variants on carotid intima-media thickness [11]. Considering the limitations of observational studies, several studies have attempted to use MR to assess the relationship between Lp(a) and CVD: Yuesong Pan and colleagues found that Lp(a) concentrations were causally associated with an increased risk of large artery stroke but a decreased risk of small vessel stroke among European individuals [12]; Helgadottir S and colleagues showed that high Lp(a) concentrations were a cause of myocardial infarction and ischemic heart disease [13]; available evidence from genetic studies supports a causal role of Lp(a) and CVD, mainly including coronary heart disease, peripheral arterial disease, aortic valve stenosis and ischemic stroke [14]. Nevertheless, these MR studies were performed in populations such as Europeans, African-Americans, and residents of Copenhagen, while limited results are available for the Chinese population. Given that Lp(a) levels are highly variable and ethnically specific [15] and that the level of Lp(a) was lower in the Han Chinese population than in Western populations [16, 17], there is a critical need for a precise characterization of the Lp(a)-associated metabolic disease risk in the Chinese population. The present study aimed to use two-sample MR to comprehensively evaluate the causal association between variants in genes affecting Lp(a) concentrations and CVD in East Asian populations.

## Methods

### Study design and data sources

**Fig. 1:**
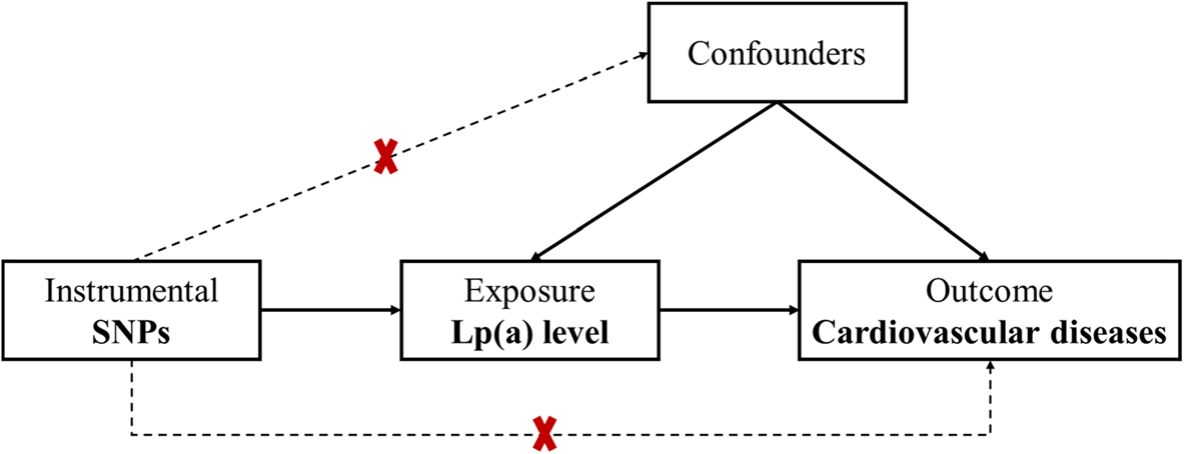
Conceptual framework for the Mendelian randomization analysis of Lp(a) and the risk of cardiovascular diseases

Two-sample MR analysis was used to assess the causal effect of Lp(a) concentrations on the risk of CVD (Fig. 1). Summary statistics for Lp(a) variants were obtained from the Cohort Study on Chronic Disease of Communities Natural Population in Beijing, Tianjin and Hebei (CHCN-BTH), which is a large, prospective, population-based chronic disease study of the Communities Natural Population initiated in 2017 to explore risk factors for major chronic diseases; the design and rationale of the study have been described previously in detail [18, 19]. Individuals were excluded if there had missing data on the key demographic variables (including age and gender), Lp(a), or CVD, or if they were of non-Han ethnicity. After screening and matching, a total of 1256 individuals with coronary heart diseases and frequency-matched controls were ultimately included in the CHCN-BTH, and each was genotyped. Patients were eligible if they had been diagnosed with CVD in the hospital, and controls were residents of the same communities who did not have CVD. Data on associations between single-nucleotide polymorphisms (SNPs) and CVD were obtained from a recently published online report on the platform of genome-wide association studies (GWAS, https://www.ebi.ac.uk/gwas/) [20]. All data in this analysis were based on subjects of Han Chinese ethnicity.

The CHCN-BTH study was approved by the Ethics Committee of the Center of Disease Control (IRB2017–003, CYCDPCIRB-20170830-1) and Capital Medical University (2018SY81), and written informed consent was obtained from all individuals prior to the baseline survey. The data on associations between SNPs and outcomes were based on publicly shared databases, and no additional participant ethical consent is required.

### Laboratory procedures

Serum Lp(a) levels were determined by using a particle-enhanced turbidimetric immunoassay, Lp(a) Latex [DAIICHI] (Sekisui Diagnostic Ltd., Japan). This assay uses a highly specific monoclonal antibody and has high sensitivity. Measurements are linear in the range of 1–100 mg/dL. Three independent calibrators and two quality control materials were used to control for apo(a) size heterogeneity. The intra-assay coefficients of variation were lower than 5%, and inter-assay coefficients of variation were lower than 10%.

### Selection of genetic variants

Lp(a)-related candidate genes were selected based on the National Human Genome Research Institute (NHGRI) and published GWAS data. The present study used the SNP function prediction (https://snpinfo.niehs.nih.gov/) to assess the predicted functional effects of the selected SNPs. Then, linkage disequilibrium (LD) analysis with an *R*^2^ threshold of 0.8 was performed with Haploview software (https://www.broad.mit.edu/haploview/haploview) for tagging SNP selection. Under these criteria, a total of 29 SNP loci were selected in the present study for further evaluation: four SNPs (rs1018234, rs2048327, rs2457574, rs520829, and rs641990) in SLC22A3, 2 SNPs (rs14224 and rs783147) in PLG, 3 SNPs (rs1510224, rs2504921, and rs3127599) in LPAL2, 1 SNP (rs2140650) in MAP3K4, 4 SNPs (rs3798220, rs6415084, rs7765781, and rs7770628) in LPA, 2 SNPs (rs429358 and rs7412) in APOE, 2 SNPs (rs505151 and rs662145) in PCSK9, 5 SNPs (rs5925, rs5927, rs5929, rs5930, and rs688) in LDLR, and 5 SNPs (rs117052562, rs1406888, rs41269133, rs56393506 and rs9457778) in intergenic regions.

### SNP genotyping

A total of 1256 blood samples were collected and centrifuged at 4000 r/min for 5 min. After centrifugation, the samples were stored at − 80 °C. Genomic DNA was extracted from 300 μL blood samples using a CWE9600 Blood DNA Kit (Kangwei Shiji Biotechnology Co., Ltd.). The Sequenom MassARRAY platform (San Diego, Calif) was applied for the candidate SNP genotyping.

### Outcomes

Summary statistics for the associations of each SNP with any cardiovascular diseases in the East Asian population were obtained from a previously published GWAS. Three large observational studies of the disease traits in the Japanese population were included, with sample sizes of 212,453 [21], 128,184 [22] and 162,255 [23], respectively. This study calculated the power using Mendelian Randomization with a Binary Outcome by PASS software (https://www.ncss.com/software/pass/). The results showed that these sample size can make sure this study’s statistical power was at least 0.83. Finally, the summarized data for the associations of the 13 SNPs for Lp(a) level with any congestive heart failure (CHF) [21], ischemic stroke (IS) [21], atrial fibrillation (AF) [22], arrhythmia [21], left ventricular mass index (LVMI) [23], left ventricular internal dimension in diastole (LVDd) [23], and left ventricular internal dimension in systole (LVDs) [23] were derived; these data are presented in Additional File 1 Table S1. The clinical information was obtained from medical records, and all the clinical end points were diagnosed by physicians.

### Statistical analyses

Effect sizes of the SNP and Lp(a) levels were estimated in the present study population using multiple linear regression under an additive model with adjustment for age and sex. Two-sample MR analyses were performed to compute estimates of Lp(a)-outcome (CHF, IS, AF, arrhythmia, LVMI, LVDd, and LVDs) associations using summarized data on the SNP-Lp(a) level and SNP-outcome associations. The MR-Egger regression method was used to evaluate the potential pleiotropic effects, and also a leave-one-out analysis was performed to investigate the influence of outlying or pleiotropic genetic variants. Heterogeneity across all instrumental SNPs was estimated by Cochran’s Q statistic. All analyses were carried out using R software (version 4.0.2, https://www.R-project.org/).

## Results

### Participant characteristics

Table 1 presents a summary of participant characteristics. The median Lp(a) concentration was 28.04 mg/dL. There were more female (*n* = 1028) than male participants (*n* = 228), and the median age of the participants was 67 for males and 62 for females. The participants were slightly overweight [median body mass index (BMI) 25.37 kg/m^2^]. Of the 1256 individuals in the sample, 22.7% were diagnosed with diabetes, 56.37% with hypertension, and 53.42% had hyperlipidemia. The median values for glucose, triglyceride, total cholesterol, high-density lipoprotein cholesterol and low-density lipoprotein cholesterol were 5.60 mmol/L, 1.40 mmol/L, 5.22 mmol/L, 1.41 mmol/L, and 3.02 mmol/L, respectively.

**Table 1:** Characteristics of the study population

Characteristics	Male	Female	All
*N* (%)	228 (18.15)	1028 (81.85)	1256 (100)
Lp(a), mg/dL	24.77 (7.95–47.03)	28.81 (9.91–47.17)	28.04 (9.45–47.17)
Age, years	67.00 (62.00–71.00)	62.00 (54.00–67.00)	63.00 (56.00–68.00)
BMI, kg/m^2^	25.38 (24.81–27.61)	25.37 (23.27–27.64)	25.37 (23.67–27.63)
Education, *n* (%)			
Primary	47 (20.61)	268 (26.07)	315 (25.08)
Secondary	117 (51.32)	431 (41.93)	548 (43.63)
Senior	37 (16.23)	207 (20.14)	244 (19.43)
Undergraduate	27 (11.84)	122 (11.87)	149 (11.86)
Physical activity, *n* (%)			
5–7 d/w	173 (75.88)	718 (69.84)	891 (70.94)
1–4 d/w	31 (13.60)	163 (15.86)	194 (15.45)
< 1 d/w	24 (10.53)	147 (14.30)	171 (13.61)
Current smoking, *n* (%)	86 (37.72)	49 (4.77)	135 (10.75)
Alcohol, *n* (%)	131 (57.46)	133 (12.94)	264 (21.02)
Glucose, mmol/L	5.80 (5.30–6.50)	5.60 (5.10–6.40)	5.60 (5.20–6.40)
TG, mmol/L	1.30 (1.00–1.85)	1.40 (1.00–1.90)	1.40 (1.00–1.90)
TC, mmol/L	4.89 (4.13–5.58)	5.29 (4.61–6.04)	5.22 (4.52–5.98)
HDL-C, mmol/L	1.26 (1.06–1.47)	1.46 (1.24–1.74)	1.41 (1.20–1.71)
LDL-C, mmol/L	2.76 (2.19–3.32)	3.06 (2.48–3.69)	3.02 (2.44–3.63)
Diabetes, *n* (%)	51 (22.37)	235 (22.86)	286 (22.77)
Hypertension, *n* (%)	157 (68.86)	551 (53.60)	708 (56.37)
Hyperlipidemia, *n* (%)	122 (53.51)	549 (53.40)	671 (53.42)

Values are *n* (%) for categorical variables or median (interquartile range) for continuous variables. *TG* Triglycerides, *TC* Total cholesterol, *HDL-C* high-density lipoprotein cholesterol, *LDL-C* low-density lipoprotein cholesterol

### Identification of Lp(a)-associated genetic variants

Only one of the 29 SNPs showed significant deviation from the Hardy-Weinberg equilibrium (HWE) (rs662145, *P* = 0.03321). After adjusting for age and sex, 13 SNPs were significantly associated with Lp(a) levels (*P* < 0.05): four SNPs in SLC22A3, 3 SNPs in LPA, 1 SNP in APOE, and 5 SNPs in undefined pathways (Table 2).

**Table 2:** GWAS identified SNPs Associated with Lp(a) Level

SNP	gene	Genomic Coordinate	EA/OA	MAF	HWE(*P*)	*β*	SE	*P* Value
rs1018234	SLC22A3	Chr6: 160375026	T/C	0.3798	0.76208	−0.10526	0.04359	0.0158924620
rs117052562	–	Chr6: 160689582	A/G	0.0510	0.98824	0.34649	0.09508	0.0002792119
rs1406888	–	Chr6: 160670561	C/T	0.4591	0.84080	−0.11589	0.04178	0.0056265889
rs14224	PLG	Chr6: 160716747	C/T	0.4669	0.75426	−0.03305	0.04251	0.4370000000
rs1510224	LPAL2	Chr6: 160473846	C/T	0.0739	0.52604	0.03701	0.08178	0.6509000000
rs2048327	SLC22A3	Chr6: 160442500	C/T	0.4502	0.99758	0.10507	0.04207	0.0126382227
rs2140650	MAP3K4	Chr6: 161087406	G/A	0.3442	0.89826	0.04999	0.04449	0.2614000000
rs2457574	SLC22A3	Chr6: 160447669	G/A	0.4542	0.99196	0.10907	0.04193	0.0094065915
rs2504921	LPAL2	Chr6: 160472974	T/G	0.3637	0.57725	0.07793	0.04293	0.0697000000
rs3127599	LPAL2	Chr6: 160486102	T/C	0.1507	0.86040	−0.00228	0.05921	0.9694000000
rs3798220	LPA	Chr6: 160540105	C/T	0.0748	0.92082	0.0249	0.08039	0.7568000000
rs41269133	–	Chr6: 160666831	C/T	0.2293	0.98939	−0.54028	0.04781	2.92E-28
rs429358	APOE	Chr19: 44908684	C/T	0.0829	0.97570	−0.19333	0.07645	0.0115732887
rs505151	PCSK9	Chr1: 55063514	G/A	0.0717	0.98202	0.10972	0.08146	0.1783000000
rs520829	SLC22A3	Chr6: 160346873	G/T	0.3157	0.72279	−0.11847	0.04555	0.0094079135
rs56393506	–	Chr6: 160668275	T/C	0.1778	0.81177	0.71204	0.05135	9.08E-41
rs5925	LDLR	Chr19: 11120205	C/T	0.2178	0.36628	0.07037	0.05181	0.1746000000
rs5927	LDLR	Chr19: 11123265	A/G	0.0518	0.93586	−0.09713	0.09432	0.3033000000
rs5929	LDLR	Chr19: 11116124	T/C	0.3062	0.97795	−0.01452	0.04569	0.7506000000
rs5930	LDLR	Chr19: 11113589	A/G	0.3665	0.16534	−0.08394	0.04471	0.0607000000
rs6415084	LPA	Chr6: 160559298	T/C	0.1663	0.67734	0.62622	0.05342	3.51E-30
rs641990	SLC22A3	Chr6: 160358102	A/G	0.3945	0.58243	−0.07539	0.04388	0.0860000000
rs662145	PCSK9	Chr1: 55064155	C/T	0.1231	0.03321	0.12342	0.06155	0.0451562913
rs688	LDLR	Chr19: 11116926	T/C	0.1618	0.72698	0.05544	0.05762	0.3362000000
rs7412	APOE	Chr19: 44908822	T/C	0.0809	0.15342	−0.14293	0.07983	0.0736000000
rs7765781	LPA	Chr6: 160586464	C/G	0.4021	0.17321	−0.41375	0.04012	5.45E-24
rs7770628	LPA	Chr6: 160597142	C/T	0.1835	0.95012	0.70012	0.05102	5.09E-40
rs783147	PLG	Chr6: 160716958	G/A	0.4980	0.51151	−0.0018	0.04264	0.9664000000
rs9457778	–	Chr6: 159932259	C/T	0.3705	0.22391	0.09361	0.04241	0.0274770059

*SNP* single nucleotide polymorphism, *EA/OA* effect allele/ other allele, *MAF* minor allele frequency, *HWE(P) P* value of Hardy-Weinberg equilibrium, *SE* standard error; sex and age were included into models for adjustment

### Causal association of Lp(a) with CVD

**Fig. 2:**
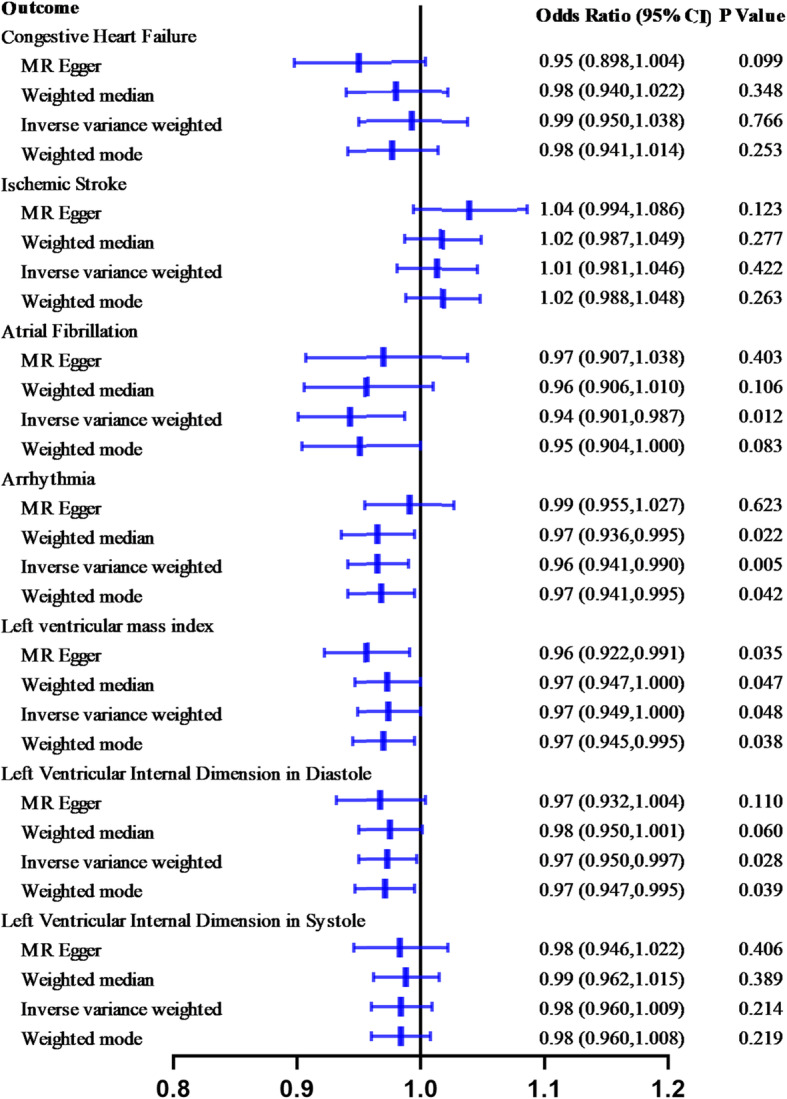
Causal estimates of genetically predicted Lp(a) level in cardiovascular diseases

**Fig. 3:**
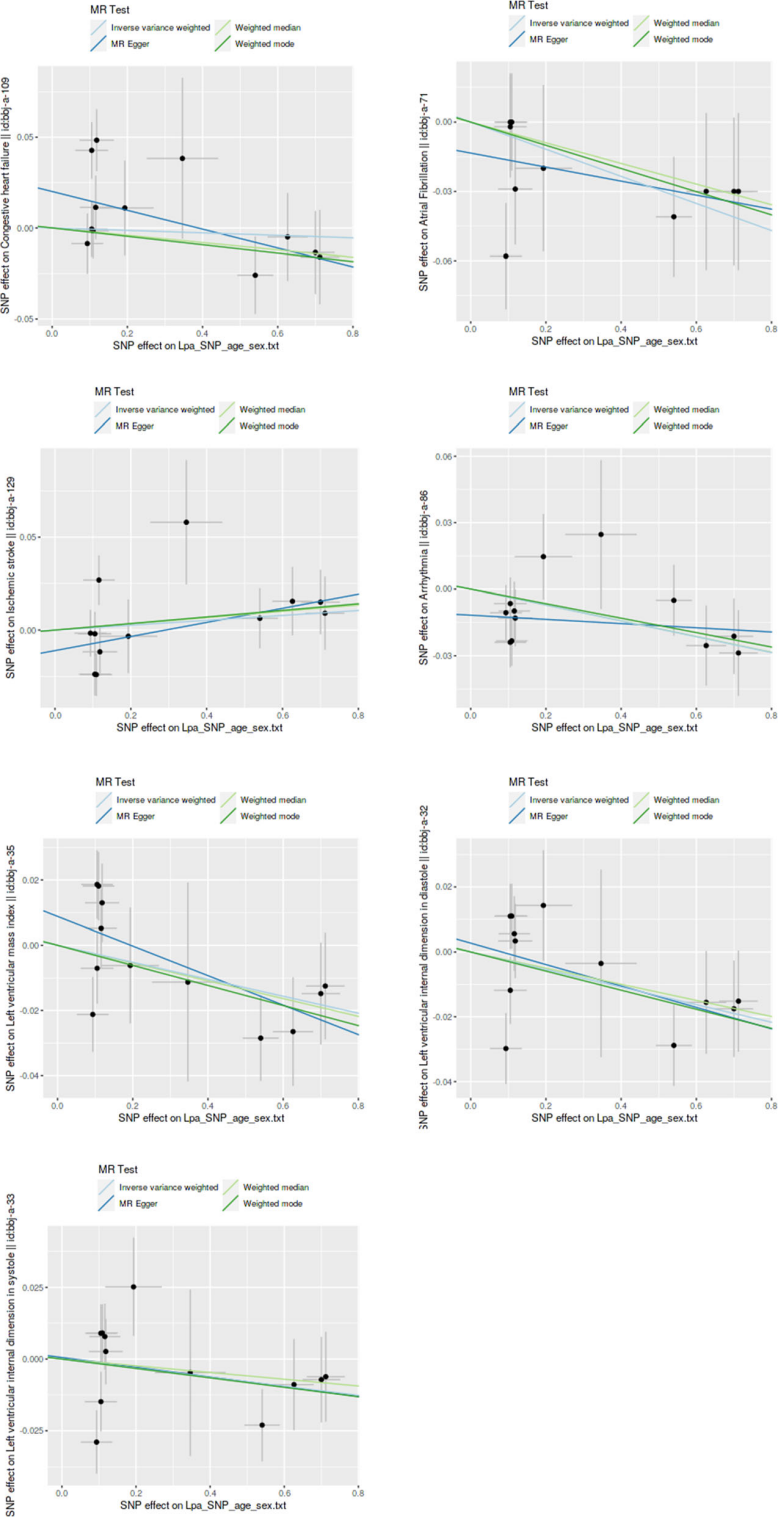
1 Associations of Lp(a) variants with Congestive Heart Failure in different methods. Fig. 3–2. Associations of Lp(a) variants with Ischemic Stroke in different methods. Fig. 3–3. Associations of Lp(a) variants with Atrial Fibrillation in different methods. Fig. 3–4. Associations of Lp(a) variants with Arrhythmia in different methods. Fig. 3–5. Associations of Lp(a) variants with Left Ventricular Mass Index in different methods. Fig. 3–6. Associations of Lp(a) variants with Left Ventricular Internal Dimension in Diastole in different methods. Fig. 3–7. Associations of Lp(a) variants with Left Ventricular Internal Dimension in Systole in different methods

Figure 2 summarizes the main MR results exploring the causal association of Lp(a) with CVD. The results support a causal relationship between Lp(a) levels and atrial fibrillation [odds ratio (OR), 0.94; 95% confidence interval (95%CI), 0.901–0.987; *P* = 0.012], arrhythmia (OR, 0.96; 95%CI, 0.941–0.990; *P* = 0.005), the left ventricular mass index (OR, 0.97; 95%CI, 0.949–1.000; *P* = 0.048), and the left ventricular internal dimension in diastole (OR, 0.97; 95%CI, 0.950–0.997; *P* = 0.028) in the inverse-variance weighted (IVW) analysis. In contrast, no significant association of genetically elevated Lp(a) levels with congestive heart failure (OR, 0.99; 95% CI, 0.950–1.038; *P* = 0.766), ischemic stroke (OR, 1.01; 95%CI, 0.981–1.046; *P* = 0.422), and left ventricular internal dimension in systole (OR, 0.98; 95%CI, 0.960–1.009; *P* = 0.214) was observed. Three additional MR methods yielded consistent results (Fig. 2, Fig. 3 -1 through Fig. 3–7).

MR-Egger regression suggested no evidence of horizontal pleiotropy and no substantial heterogeneity among individual SNPs (Table 3). The results of leave-one-out sensitivity analysis showed that the association between Lp(a) levels and CHF, IS, AF, arrhythmia, LVMI, LVDd, and LVDs was not substantially driven by any individual SNP (Additional File 1 Fig. 1–1 through 1–7). Single SNP analysis for Lp(a) on CVD subtypes is also displayed in Additional File 1 Table S2 and Fig. 2–1 through Fig. 2–7.

**Table 3:** Horizontal pleiotropy

Outcome	Horizontal pleiotropy			Heterogeneity statistics	
	beta	se	*P* value	*Q* value-Egger	*Q* value-IVW
Congestive heart failure	0.0200	0.0092	0.0541	0.2219	0.0570
Ischemic stroke	−0.0110	0.0073	0.1600	0.1650	0.0956
Atrial fibrillation	−0.0130	0.0120	0.2950	0.7055	0.6646
Arrhythmia	−0.0120	0.0060	0.0815	0.7450	0.4824
Left ventricular mass index	0.0088	0.0062	0.1870	0.2577	0.1863
Left ventricular internal dimension in diastole	0.0027	0.0063	0.6750	0.1659	0.2105
Left ventricular internal dimension in systole	0.00055	0.0066	0.9350	0.1254	0.1737

## Discussion

Using a two-sample MR analysis design to evaluate the causal association between variants in genes encoding Lp(a) concentrations and CVD in East Asian populations, this study observed an association between genetically elevated Lp(a) and decreased risk of atrial fibrillation and arrhythmia, as well as the values of the left ventricular mass index and the left ventricular internal dimension in diastole. In contrast, no significant association was found between genetically elevated Lp(a) and the risk of congestive heart failure, the risk of ischemic stroke, and the value of the left ventricular internal dimension in systole. The results were robust in sensitivity analysis with different instruments and statistical models.

Consistent with Steffen BT and colleagues’ findings [24], no significant association between Lp(a) and heart failure was observed in the Han Chinese population. However, the results concerning heart failure were less consistent with previous observational studies [25, 26]. Elevated Lp(a) levels were found to be associated with an increased risk of incident heart failure hospitalization in the Atherosclerosis Risk in Communities (ARIC) study, with 14,154 participants, whereas after excluding the prevalent and incident myocardial infarction, the association was no longer significant [26]. Gudbjartsson DF and colleagues’ results also indicated that the odds ratio of 1.05 (95%CI: 1.02–1.07) for heart failure with a 50 nmol/L increase in Lp(a) lost significance after controlling for Lp(a) concentration (OR: 1.01, 95%CI: 0.98–1.04) [27]. Using instrumental variable analysis, Kamstrup PR and colleagues indicated that each 10-fold increase in Lp(a) levels was associated with a genetic relative risk for heart failure of 1.18 (95%CI: 1.04–1.34) [25]. Racial differences may be one of the reasons for the inconsistent findings, as it is well known that Lp(a) concentrations are highly variable with race/ethnicity, with the lowest Lp(a) levels occurring in Chinese individuals [15, 28]. When Lp(a) levels were below the 66th percentile (8 to 19 mg/dL), their effect on cardiovascular risk was not statistically significant. However, based on the data of the Cohort Study on Chronic Diseases of the General Community Population in the Beijing-Tianjin-Hebei Region (CHCN-BTH Study, Registration number: ChiCTR1900024725) project [18], the median level of Lp(a) was 11.9 mg/dL (IQR 5.9 to 23.7 mg/dL), which was in the statistically nonsignificant range in the study by Kamstrup PR and colleagues.

The findings revealed a null association between genetically predicted Lp(a) levels and ischemic stroke, which is similar to the previous results in a Mendelian randomization study published by Pan et al. [12] However, such associations were less consistent in some previous observational and MR studies [29]. In the Copenhagen City Heart Study and Copenhagen General Population Study population [30], subjects with high Lp(a) levels were associated with an increased risk of ischemic stroke both observationally (hazard ratio: 1.20; 95%CI: 1.13–1.28) and causally (hazard ratio: 1.20; 95%CI: 1.02–1.43). Gudbjartsson DF and colleagues found that the odds ratio of 1.03 (95%CI: 1.00–1.07) for stroke for a 50 nmol/L increase in Lp(a) lost significance when KIV-2 repeats were used (OR: 1.01; 95%CI: 0.98–1.04) [27]. A recent retrospective case-control study indicated that elevated Lp(a) was significantly associated with increased ischemic stroke risk (OR = 2.03, 2.36, and 2.03 for quartiles 2, 3 and 4, respectively, vs. quartile 1) in Han Chinese individuals, especially among men and younger patients [31]. Some of the factors may have contributed to the differential findings among the present study and previous studies. First, Lp(a) levels had differential associations by sex and race/ethnicity [32]; differences in participant characteristics might be one of the main reasons for the discrepancy. Second, the results of the cross-sectional study might be biased by some confounders, such as myocardial infarction [26]. Furthermore, most previous studies included all stroke subtypes [13, 33–36], while the association between Lp(a) levels and the risk of different subtypes of stroke might be different; for example, elevated Lp(a) concentrations were reported to have a positive association with large artery stroke, and an inverse association with small vessel stroke. All of these may cause variable results.

Fewer studies have reported the associations of Lp(a) with AF, arrhythmia, and the left ventricular mass index. In a prospective study of 23,738 healthy middle-aged and older women, the results indicated that Lp(a) was not associated with AF in multivariable models and that there was no significant linear trend (*p* for direction = 0.63) for the risk of AF across Lp(a) quintiles [37]. Aronis KN and colleagues also reported no significant association between Lp(a) and incident AF (hazard ratio: 0.98, 95%CI: 0.82–1.17) [38]. However, in the Multi-Ethnic Study of Atherosclerosis (MESA) study, the results showed that an Lp(a) level ≥ 30 mg/dL was inversely associated with the risk of AF (hazard ratio: 0.84, 95%CI: 0.71–0.99) [39], which was consistent with the results of the present MR analysis. The reasons for the disparity of the above results may lie in the demographic differences between the samples. For example, the study by Mora S and colleagues included only women [37], and the study by Aronis KN included White and Blacks participants [38]. However, in Chinese individuals, the results showed a significant difference in both cross-sectional and MR studies. Consistent with the AF results, significant associations of Lp(a) with arrhythmia, the left ventricular mass index and the left ventricular internal dimension in diastole were also observed.

Although the mechanism inversely linking high Lp(a) levels with the risk of AF and arrhythmia remains quite unclear, a similar relationship has been reported for blood lipids, in which elevated total cholesterol, low-density lipoprotein cholesterol, and high-density lipoprotein levels were found to be associated with a low risk of AF in a meta-analysis of cohort studies [40]. Inverse associations of AF with small cholesterol-poor low-density lipoprotein cholesterol, the total number of low-density lipoprotein particles, and the total number of very-low-density lipoprotein particles were driven by the numbers of cholesterol-poor small low-density lipoprotein particles and small very-low-density lipoprotein particles rather than larger cholesterol-rich LDL particles, suggesting that there might be mechanisms beyond the direct effects of cholesterol [37]. These findings could be partially explained the consideration that Lp(a) consists of a low-density lipoprotein-like particle and contains approximately 45% cholesterol [41, 42]. Further research is needed to explore the specific mechanisms in the future.

The present study has potential implications for clinical practice. Some previous studies showed that very high Lp(a) levels (above the threshold of 60 mg/dL) had a significant association with recurrent cardiovascular events in patients with premature coronary artery disease and atherosclerotic cardiovascular disease [43]. Therefore, therapeutic strategies that specifically address the reduction of Lp(a) levels are receiving a great deal of attention in clinical practice [44]. The present study results can help clinicians manage patients with CVD by identifying the risks associated with very low Lp(a) levels in patients taking Lp(a)-lowering treatment and improving the prognostic assessment. These findings could be used to provide evidence for the design of further research towards the treatment and prevention of CVD in the Chinese population.

### Strengths and limitations

An advantage of this study was that the present study evaluated the associations of genetically predicted Lp(a) levels with CVD in the Han Chinese population, and the results have been limited thus far. Since the results vary among ethnic groups, this study provides evidence to enable the precise treatment of the Chinese population. Through the use of MR, the potential bias can be greatly reduced, and reverse causation can also be avoided [45]. However, the results were limited to the East Asian population, which may limit the generalizability of these findings to other populations. Some of the individuals were in a chronic disease registry, considering that Lp(a) is mainly determined by the LPA gene, there might be a slight influence on the results. In addition, due to financial constraints, additional Lp(a) related loci could not be detected; thus, the present study could not validate the relationship of Lp(a) with coronary heart disease and some other CVD subtypes. This will be pursued further in future research.

## Conclusions

In conclusion, the present study provided evidence that genetically elevated Lp(a) was inversely associated with atrial fibrillation, arrhythmia, the left ventricular mass index and the left ventricular internal dimension in diastole but not with congestive heart failure, ischemic stroke, or the left ventricular internal dimension in systole. These results can help clinicians manage patients with CVD by identifying the risks associated with very low Lp(a) levels in patients taking Lp(a)-lowering treatment and improving the prognostic assessment. Further research should address the mechanism underlying these results and determine whether genetically elevated Lp(a) increases the risk of coronary heart disease or other CVD subtypes.

## Supplementary Information

**Additional file 1: Table S1**. Genetic association of Lp(a) level related 13 genetic variants with cardiovascular disease subtypes. **Table S2**. MR base single SNP. Figure 1**–1**. MR leave-one-out sensitivity analysis for Lp(a) on Congestive Heart Failure. Figure 1**–2**. MR leave-one-out sensitivity analysis for Lp(a) on Ischemic Stroke. Figure 1**–3**. MR leave-one-out sensitivity analysis for Lp(a) on Atrial Fibrillation. Figure 1**–4**. MR leave-one-out sensitivity analysis for Lp(a) on Arrhythmia. Figure 1**–5**. MR leave-one-out sensitivity analysis for Lp(a) on Left Ventricular Mass Index. Figure 1**–6**. MR leave-one-out sensitivity analysis for Lp(a) on Left Ventricular Internal Dimension in Diastole. Figure 1**–7**. MR leave-one-out sensitivity analysis for Lp(a) on Left Ventricular Internal Dimension in Systole**.** Figure 2**–1**. Single SNP analysis for Lp(a) on Congestive Heart Failure. Figure 2**–2**. Single SNP analysis for Lp(a) on Ischemic Stroke. Figure 2**–3**. Single SNP analysis for Lp(a) on Atrial Fibrillation. Figure 2**–4**. Single SNP analysis for Lp(a) on Arrhythmia. Figure 2**–5**. Single SNP analysis for Lp(a) on Left Ventricular Mass Index. Figure 2**–6**. Single SNP analysis for Lp(a) on Left Ventricular Internal Dimension in Diastole. Figure 2**–7**. Single SNP analysis for Lp(a) on Left Ventricular Internal Dimension in Systole

## Data Availability

The summary statistics for Lp(a) variants used during the current study are available from the corresponding author on reasonable request. Data on associations of SNPs with CVD were obtained from the recently published online on the platform of GWAS.

## Abbreviations

Lp(a): Lipoprotein (a)
CVD: Cardiovascular disease
CHCN-BTH: The Cohort Study on Chronic Disease of Communities Natural Population in Beijing, Tianjin and Hebei
MR: Mendelian randomization
SNP: Single nucleotide polymorphism
GWAS: Genome-wide association studies
CHF: Congestive Heart Failure
IS: Ischemic Stroke
AF: Atrial Fibrillation
LVMI: Left Ventricular Mass Index
LVDd: Left Ventricular Internal Dimension in Diastole
LVDs: Left Ventricular Internal Dimension in Systole
OR: Odds ratio
CI: Confidence interval
IVW: Inverse-variance weighted

## References

[1] National Center for Cardiovascular Diseases, China (2020). Annual report on cardiovascular health and diseases in China.

[2] World Health Organization. https://www.who.int/news/item/09-12-2020-who-reveals-leading-causes-of-death-and-disability-worldwide-2000-2019 (accessed January 19, 2021).

[3] Zhao D, Liu J, Wang M, Zhang X, Zhou M (2019). Epidemiology of cardiovascular disease in China: current features and implications. Nat Rev Cardiol.

[4] The Writing Committee of the Report on Cardiovascular Health and Diseases in China. Interpretation of Report on Cardiovascular Health and Diseases in China (2019). Chin. J Cardiovasc Med, 2020.

[5] Ooi EM, Ellis KL, Barrett PHR, Watts GF, Hung J, Beilby JP (2018). Lipoprotein(a) and apolipoprotein(a) isoform size: associations with angiographic extent and severity of coronary artery disease, and carotid artery plaque. Atherosclerosis..

[6] Rallidis LS, Pavlakis G, Foscolou A, Kotakos C, Katsimardos A, Drosatos A, Zolindaki M, Panagiotakos DB (2018). High levels of lipoprotein (a) and premature acute coronary syndrome. Atherosclerosis..

[7] Verbeek R, Hoogeveen RM, Langsted A, Stiekema LCA, Verweij SL, Hovingh GK, Wareham NJ, Khaw KT, Boekholdt SM, Nordestgaard BG, Stroes ESG (2018). Cardiovascular disease risk associated with elevated lipoprotein(a) attenuates at low low-density lipoprotein cholesterol levels in a primary prevention setting. Eur Heart J.

[8] Nordestgaard BG, Chapman MJ, Ray K, Borén J, Andreotti F, Watts GF, Ginsberg H, Amarenco P, Catapano A, Descamps OS, Fisher E, Kovanen PT, Kuivenhoven JA, Lesnik P, Masana L, Reiner Z, Taskinen MR, Tokgözoglu L, Tybjærg-Hansen A, European Atherosclerosis Society Consensus Panel (2010). Lipoprotein(a) as a cardiovascular risk factor: current status. Eur Heart J.

[9] Smith GD, Ebrahim S (2003). 'Mendelian randomization': can genetic epidemiology contribute to understanding environmental determinants of disease?. Int J Epidemiol.

[10] Boras J, Ljubic S, Car N, Metelko Z, Petrovecki M, Lovrencic MV, Reiner Z (2010). Lipoprotein(a) predicts progression of carotid artery intima-media thickening in patients with type 2 diabetes: a four-year follow-up. Wien Klin Wochenschr.

[11] Kivimäki M, Magnussen CG, Juonala M, Kähönen M, Kettunen J, Loo BM, Lehtimäki T, Viikari J, Raitakari OT (2011). Conventional and Mendelian randomization analyses suggest no association between lipoprotein(a) and early atherosclerosis: the young Finns study. Int J Epidemiol.

[12] Pan Y, Li H, Wang Y, Meng X, Wang Y (2019). Causal effect of Lp(a) [lipoprotein(a)] level on ischemic stroke and Alzheimer disease: a Mendelian randomization study. Stroke..

[13] Helgadottir A, Gretarsdottir S, Thorleifsson G, Holm H, Patel RS, Gudnason T, Jones GT, van Rij AM, Eapen DJ, Baas AF, Tregouet DA, Morange PE, Emmerich J, Lindblad B, Gottsäter A, Kiemeny LA, Lindholt JS, Sakalihasan N, Ferrell RE, Carey DJ, Elmore JR, Tsao PS, Grarup N, Jørgensen T, Witte DR, Hansen T, Pedersen O, Pola R, Gaetani E, Magnadottir HB, Wijmenga C, Tromp G, Ronkainen A, Ruigrok YM, Blankensteijn JD, Mueller T, Wells PS, Corral J, Soria JM, Souto JC, Peden JF, Jalilzadeh S, Mayosi BM, Keavney B, Strawbridge RJ, Sabater-Lleal M, Gertow K, Baldassarre D, Nyyssönen K, Rauramaa R, Smit AJ, Mannarino E, Giral P, Tremoli E, de Faire U, Humphries SE, Hamsten A, Haraldsdottir V, Olafsson I, Magnusson MK, Samani NJ, Levey AI, Markus HS, Kostulas K, Dichgans M, Berger K, Kuhlenbäumer G, Ringelstein EB, Stoll M, Seedorf U, Rothwell PM, Powell JT, Kuivaniemi H, Onundarson PT, Valdimarsson E, Matthiasson SE, Gudbjartsson DF, Thorgeirsson G, Quyyumi AA, Watkins H, Farrall M, Thorsteinsdottir U, Stefansson K (2012). Apolipoprotein(a) genetic sequence variants associated with systemic atherosclerosis and coronary atherosclerotic burden but not with venous thromboembolism. J Am Coll Cardiol.

[14] Kamstrup PR (2021). Lipoprotein(a) and cardiovascular disease. Clin Chem.

[15] Lee SR, Prasad A, Choi YS, Xing C, Clopton P, Witztum JL, Tsimikas S (2017). LPA gene, ethnicity, and cardiovascular events. Circulation..

[16] Cui FM, Fang F, He YM, Cai DP, He J, Yang XJ (2018). Establishing age and sex dependent upper reference limits for the plasma lipoprotein (a) in a Chinese health check-up population and according to its relative risk of primary myocardial infarction. Clin Chim Acta.

[17] Liu HH, Cao YX, Jin JL, Zhang HW, Hua Q, Li YF, Guo YL, Zhu CG, Wu NQ, Xu RX, Chen XH, Li JJ (2020). Predicting cardiovascular outcomes by baseline lipoprotein(a) concentrations: a large cohort and long-term follow-up study on real-world patients receiving percutaneous coronary intervention. J Am Heart Assoc.

[18] Cao H, Li B, Peng W, Pan L, Cui Z, Zhao W, Zhang H, Tang N, Niu K, Sun J, Han X, Wang Z, Liu K, He H, Cao Y, Xu Z, Shan A, Meng G, Sun Y, Guo C, Liu X, Xie Y, Wen F, Shan G, Zhang L (2020). Associations of long-term exposure to ambient air pollution with cardiac conduction abnormalities in Chinese adults: the CHCN-BTH cohort study. Environ Int.

[19] Cao H, Shan GL, Zhang L., An introduction of the cohort study on chronic diseases of natural population in the living community of Beijing-Tianjin-Hebei region. Chin J Evid Based Med, 2018. 18(6): p5.

[20] Hemani G, Zheng J, Elsworth B, Wade KH, Haberland V, Baird D, et al. The MR-Base platform supports systematic causal inference across the human phenome. Elife. 2018;7:e34408. 10.7554/eLife.34408. 10.7554/eLife.34408 PMC5976434 29846171

[21] Ishigaki K, Akiyama M, Kanai M, Takahashi A, Kawakami E, Sugishita H, Sakaue S, Matoba N, Low SK, Okada Y, Terao C, Amariuta T, Gazal S, Kochi Y, Horikoshi M, Suzuki K, Ito K, Koyama S, Ozaki K, Niida S, Sakata Y, Sakata Y, Kohno T, Shiraishi K, Momozawa Y, Hirata M, Matsuda K, Ikeda M, Iwata N, Ikegawa S, Kou I, Tanaka T, Nakagawa H, Suzuki A, Hirota T, Tamari M, Chayama K, Miki D, Mori M, Nagayama S, Daigo Y, Miki Y, Katagiri T, Ogawa O, Obara W, Ito H, Yoshida T, Imoto I, Takahashi T, Tanikawa C, Suzuki T, Sinozaki N, Minami S, Yamaguchi H, Asai S, Takahashi Y, Yamaji K, Takahashi K, Fujioka T, Takata R, Yanai H, Masumoto A, Koretsune Y, Kutsumi H, Higashiyama M, Murayama S, Minegishi N, Suzuki K, Tanno K, Shimizu A, Yamaji T, Iwasaki M, Sawada N, Uemura H, Tanaka K, Naito M, Sasaki M, Wakai K, Tsugane S, Yamamoto M, Yamamoto K, Murakami Y, Nakamura Y, Raychaudhuri S, Inazawa J, Yamauchi T, Kadowaki T, Kubo M, Kamatani Y (2020). Large-scale genome-wide association study in a Japanese population identifies novel susceptibility loci across different diseases. Nat Genet.

[22] Low SK, Takahashi A, Ebana Y, Ozaki K, Christophersen IE, Ellinor PT (2017). Identification of six new genetic loci associated with atrial fibrillation in the Japanese population. Nat Genet.

[23] Kanai M, Akiyama M, Takahashi A, Matoba N, Momozawa Y, Ikeda M, Iwata N, Ikegawa S, Hirata M, Matsuda K, Kubo M, Okada Y, Kamatani Y (2018). Genetic analysis of quantitative traits in the Japanese population links cell types to complex human diseases. Nat Genet.

[24] Steffen BT, Duprez D, Bertoni AG, Guan W, Tsai MY (2018). Lp(a) [lipoprotein(a)]-related risk of heart failure is evident in whites but not in other racial/ethnic groups. Arterioscler Thromb Vasc Biol.

[25] Kamstrup PR, Nordestgaard BG (2016). Elevated lipoprotein(a) levels, LPA risk genotypes, and increased risk of heart failure in the general population. JACC Heart Fail.

[26] Agarwala A, Pokharel Y, Saeed A, Sun W, Virani SS, Nambi V, Ndumele C, Shahar E, Heiss G, Boerwinkle E, Konety S, Hoogeveen RC, Ballantyne CM (2017). The association of lipoprotein(a) with incident heart failure hospitalization: atherosclerosis risk in communities study. Atherosclerosis..

[27] Gudbjartsson DF, Thorgeirsson G, Sulem P, Helgadottir A, Gylfason A, Saemundsdottir J, Bjornsson E, Norddahl GL, Jonasdottir A, Jonasdottir A, Eggertsson HP, Gretarsdottir S, Thorleifsson G, Indridason OS, Palsson R, Jonasson F, Jonsdottir I, Eyjolfsson GI, Sigurdardottir O, Olafsson I, Danielsen R, Matthiasson SE, Kristmundsdottir S, Halldorsson BV, Hreidarsson AB, Valdimarsson EM, Gudnason T, Benediktsson R, Steinthorsdottir V, Thorsteinsdottir U, Holm H, Stefansson K (2019). Lipoprotein(a) concentration and risks of cardiovascular disease and diabetes. J Am Coll Cardiol.

[28] Paré G, Çaku A, McQueen M, Anand SS, Enas E, Clarke R, Boffa MB, Koschinsky M, Wang X, Yusuf S, On behalf of the INTERHEART Investigators (2019). Lipoprotein(a) levels and the risk of myocardial infarction among 7 ethnic groups. Circulation..

[29] Pearson K, Rodriguez F (2020). Lipoprotein(a) and cardiovascular disease prevention across diverse populations. Cardiol Ther.

[30] Langsted A, Nordestgaard BG, Kamstrup PR (2019). Elevated lipoprotein(a) and risk of ischemic stroke. J Am Coll Cardiol.

[31] Fu H, Zhang D, Zhu R, Cui L, Qiu L, Lin S, Peng B (2020). Association between lipoprotein(a) concentration and the risk of stroke in the Chinese Han population: a retrospective case-control study. Ann Transl Med.

[32] Brandt EJ, Mani A, Spatz ES, Desai NR, Nasir K. Lipoprotein(a) levels and association with myocardial infarction and stroke in a nationally representative cross-sectional US cohort. J Clin Lipidol. 2020; 14(5): 695–706.e4. 10.1016/j.jacl.2020.06.010 PMC7641964 32739333

[33] Emdin CA, Khera AV, Natarajan P, Klarin D, Won HH, Peloso GM, Stitziel NO, Nomura A, Zekavat SM, Bick AG, Gupta N, Asselta R, Duga S, Merlini PA, Correa A, Kessler T, Wilson JG, Bown MJ, Hall AS, Braund PS, Samani NJ, Schunkert H, Marrugat J, Elosua R, McPherson R, Farrall M, Watkins H, Willer C, Abecasis GR, Felix JF, Vasan RS, Lander E, Rader DJ, Danesh J, Ardissino D, Gabriel S, Saleheen D, Kathiresan S, CHARGE–Heart Failure Consortium, CARDIoGRAM Exome Consortium (2016). Phenotypic characterization of genetically lowered human lipoprotein(a) levels. J Am Coll Cardiol.

[34] Erqou S, Kaptoge S, Perry PL, Di Angelantonio E, Thompson A, White IR, Emerging Risk Factors Collaboration (2009). Lipoprotein(a) concentration and the risk of coronary heart disease, stroke, and nonvascular mortality. JAMA..

[35] Virani SS, Brautbar A, Davis BC, Nambi V, Hoogeveen RC, Sharrett AR, Coresh J, Mosley TH, Morrisett JD, Catellier DJ, Folsom AR, Boerwinkle E, Ballantyne CM (2012). Associations between lipoprotein(a) levels and cardiovascular outcomes in black and white subjects: the atherosclerosis risk in communities (ARIC) study. Circulation..

[36] Arora P, Kalra R, Callas PW, Alexander KS, Zakai NA, Wadley V, Arora G, Kissela BM, Judd SE, Cushman M (2019). Lipoprotein(a) and risk of ischemic stroke in the REGARDS study. Arterioscler Thromb Vasc Biol.

[37] Mora S, Akinkuolie AO, Sandhu RK, Conen D, Albert CM (2014). Paradoxical association of lipoprotein measures with incident atrial fibrillation. Circ Arrhythm Electrophysiol.

[38] Aronis KN, Zhao D, Hoogeveen RC, Alonso A, Ballantyne CM, Guallar E (2017). Associations of lipoprotein(a) levels with incident atrial fibrillation and ischemic stroke: the ARIC (atherosclerosis risk in communities) study. J Am Heart Assoc.

[39] Garg PK, Guan W, Karger AB, Steffen BT, O'Neal W, Heckbert SR, Michos ED, Tsai MY (2020). Lp(a) (lipoprotein [a]) and risk for incident atrial fibrillation: multi-ethnic study of atherosclerosis. Circ Arrhythm Electrophysiol.

[40] Guan B, Li X, Xue W, Tse G, Waleed KB, Liu Y, et al. Blood lipid profiles and risk of atrial fibrillation: A systematic review and meta-analysis of cohort studies. J Clin Lipidol. 2020; 14(1): 133–142.e3. 10.1016/j.jacl.2019.12.002 31926850

[41] Kronenberg F, Lingenhel A, Lhotta K, Rantner B, Kronenberg MF, König P, Thiery J, Koch M, von Eckardstein A, Dieplinger H (2004). Lipoprotein(a)- and low-density lipoprotein-derived cholesterol in nephrotic syndrome: impact on lipid-lowering therapy?. Kidney Int.

[42] Maranhão RC, Carvalho PO, Strunz CC, Pileggi F (2014). Lipoprotein (a): structure, pathophysiology and clinical implications. Arq Bras Cardiol.

[43] Gragnano F, Fimiani F, Di Maio M, Cesaro A, Limongelli G, Cattano D (2019). Impact of lipoprotein(a) levels on recurrent cardiovascular events in patients with premature coronary artery disease. Intern Emerg Med.

[44] Cesaro A, Schiavo A, Moscarella E, Coletta S, Conte M, Gragnano F, Fimiani F, Monda E, Caiazza M, Limongelli G, D’Erasmo L, Riccio C, Arca M, Calabrò P (2021). Lipoprotein(a): a genetic marker for cardiovascular disease and target for emerging therapies. J Cardiovasc Med (Hagerstown).

[45] Smith GD, Ebrahim S (2004). Mendelian randomization: prospects, potentials, and limitations. Int J Epidemiol.

