# Supplementary material for: Association of Lipoprotein (a) variants with risk of cardiovascular disease: a Mendelian randomization study
Source: Lipids Health Dis. 2021 Jun 1;20:57. doi: 10.1186/s12944-021-01482-0 (PMC8170931; doi:10.1186/s12944-021-01482-0)
Supplement: Supplementary file 1 — Additional file 1: Table S1. Genetic association of Lp(a) level related 13 genetic variants with cardiovascular disease subtypes. Table S2. MR base single SNP. Figure 1–1. MR leave-one-out sensitivity analysis for Lp(a) on Congestive Heart Failure. Figure 1–2. MR leave-one-out sensitivity analysis for Lp(a) on Ischemic Stroke. Figure 1–3. MR leave-one-out sensitivity analysis for Lp(a) on Atrial Fibrillation. Figure 1–4. MR leave-one-out sensitivity analysis for Lp(a) on Arrhythmia. Figure 1–5. MR leave-one-out sensitivity analysis for Lp(a) on Left Ventricular Mass Index. Figure 1–6. MR leave-one-out sensitivity analysis for Lp(a) on Left Ventricular Internal Dimension in Diastole. Figure 1–7. MR leave-one-out sensitivity analysis for Lp(a) on Left Ventricular Internal Dimension in Systole. Figure 2–1. Single SNP analysis for Lp(a) on Congestive Heart Failure. Figure 2–2. Single SNP analysis for Lp(a) on Ischemic Stroke. Figure 2–3. Single SNP analysis for Lp(a) on Atrial Fibrillation. Figure 2–4. Single SNP analysis for Lp(a) on Arrhythmia. Figure 2–5. Single SNP analysis for Lp(a) on Left Ventricular Mass Index. Figure 2–6. Single SNP analysis for Lp(a) on Left Ventricular Internal Dimension in Diastole. Figure 2–7. Single SNP analysis for Lp(a) on Left Ventricular Internal Dimension in Systole [file 12944_2021_1482_MOESM1_ESM.docx]

Table S1. Genetic association of Lp(a) level related 13 genetic variants with cardiovascular disease subtypes

| SNPs | EA/OA | Congestive Heart Failure | | |  | Ischemic Stroke | | |  | Atrial Fibrillation | | |  | Arrhythmia | | |
| --- | --- | --- | --- | --- | --- | --- | --- | --- | --- | --- | --- | --- | --- | --- | --- | --- |
|  |  | beta | se | *P* value |  | beta | se | *P* value |  | beta | se | *P* value |  | beta | se | *P* value |
| rs1018234 | T/C | -0.04271 | 0.01560 | 0.00619 |  | 0.00202 | 0.01187 | 0.86498 |  | 0.0020 | 0.0220 | 0.92670 |  | 0.00644 | 0.01168 | 0.58158 |
| rs117052562 | A/G | 0.03828 | 0.04422 | 0.38668 |  | 0.05802 | 0.03355 | 0.08379 |  | - | - | - |  | 0.02466 | 0.03357 | 0.46258 |
| rs1406888 | C/T | -0.01134 | 0.01755 | 0.51828 |  | -0.02683 | 0.01334 | 0.04431 |  | - | - | - |  | 0.00978 | 0.01314 | 0.45671 |
| rs2048327 | C/T | -0.00062 | 0.01509 | 0.96736 |  | -0.02384 | 0.01148 | 0.03784 |  | 0 | 0.0210 | 0.99950 |  | -0.02392 | 0.01131 | 0.03436 |
| rs2457574 | G/A | -0.00155 | 0.01509 | 0.91832 |  | -0.02405 | 0.01148 | 0.03614 |  | 0 | 0.0210 | 0.98820 |  | -0.02329 | 0.01131 | 0.03938 |
| rs41269133 | C/T | 0.02594 | 0.02126 | 0.22236 |  | -0.00634 | 0.01614 | 0.69438 |  | 0.0410 | 0.0260 | 0.11660 |  | 0.00500 | 0.01596 | 0.75411 |
| rs429358 | C/T | -0.01100 | 0.02607 | 0.67296 |  | 0.00333 | 0.01991 | 0.86718 |  | 0.0200 | 0.0360 | 0.57810 |  | -0.01463 | 0.01931 | 0.44871 |
| rs520829 | G/T | -0.04833 | 0.01707 | 0.00465 |  | 0.01175 | 0.01299 | 0.36574 |  | 0.0290 | 0.0240 | 0.23430 |  | 0.01299 | 0.01279 | 0.30974 |
| rs56393506 | T/C | -0.01595 | 0.02588 | 0.53785 |  | 0.00908 | 0.01970 | 0.64471 |  | -0.0300 | 0.0340 | 0.38010 |  | -0.02875 | 0.01937 | 0.13782 |
| rs6415084 | T/C | -0.00493 | 0.02418 | 0.83851 |  | 0.01553 | 0.01841 | 0.39909 |  | -0.0300 | 0.0340 | 0.38530 |  | -0.02542 | 0.01810 | 0.16026 |
| rs7765781 | C/G | 0.01183 | 0.01628 | 0.46747 |  | -0.03260 | 0.01237 | 0.00840 |  | 0.0020 | 0.0220 | 0.93120 |  | -0.00515 | 0.01220 | 0.67259 |
| rs7770628 | C/T | -0.01343 | 0.02280 | 0.55586 |  | 0.01507 | 0.01736 | 0.38547 |  | -0.0300 | 0.0320 | 0.35980 |  | -0.02120 | 0.01705 | 0.21378 |
| rs9457778 | C/T | -0.00861 | 0.01657 | 0.60346 |  | -0.00168 | 0.01261 | 0.89408 |  | -0.0580 | 0.0230 | 0.01351 |  | -0.01057 | 0.01239 | 0.39393 |

Table S1. Genetic association of Lp(a) level related 13 genetic variants with cardiovascular disease subtypes (continue)

| SNPs | EA/OA | Left Ventricular Mass Index | | |  | Left Ventricular Internal Dimension in Diastole | | |  | Left Ventricular Internal Dimension in Systole | | |
| --- | --- | --- | --- | --- | --- | --- | --- | --- | --- | --- | --- | --- |
|  |  | beta | se | *P* value |  | beta | se | *P* value |  | beta | se | *P* value |
| rs1018234 | T/C | 0.00704 | 0.01089 | 0.51830 |  | 0.01183 | 0.01037 | 0.25380 |  | 0.01486 | 0.01039 | 0.15270 |
| rs117052562 | A/G | -0.01129 | 0.03052 | 0.71150 |  | -0.00353 | 0.02891 | 0.90280 |  | -0.00473 | 0.02899 | 0.87050 |
| rs1406888 | C/T | -0.00519 | 0.01207 | 0.66750 |  | -0.00564 | 0.01149 | 0.62340 |  | -0.00784 | 0.01152 | 0.49630 |
| rs2048327 | C/T | 0.01861 | 0.01052 | 0.07684 |  | 0.01098 | 0.01002 | 0.27290 |  | 0.00900 | 0.01004 | 0.37020 |
| rs2457574 | G/A | 0.01812 | 0.01052 | 0.08492 |  | 0.01101 | 0.01001 | 0.27150 |  | 0.00910 | 0.01004 | 0.36460 |
| rs41269133 | C/T | 0.02849 | 0.01315 | 0.03028 |  | 0.02884 | 0.01252 | 0.02129 |  | 0.02303 | 0.01256 | 0.06674 |
| rs429358 | C/T | 0.00619 | 0.01778 | 0.72760 |  | -0.01429 | 0.01705 | 0.40200 |  | -0.02517 | 0.01708 | 0.14070 |
| rs520829 | G/T | -0.01299 | 0.01202 | 0.27990 |  | -0.00341 | 0.01145 | 0.76580 |  | -0.00264 | 0.01147 | 0.81820 |
| rs56393506 | T/C | -0.01249 | 0.01639 | 0.44620 |  | -0.01517 | 0.01561 | 0.33110 |  | -0.00616 | 0.01564 | 0.69370 |
| rs6415084 | T/C | -0.02649 | 0.01670 | 0.11270 |  | -0.01554 | 0.01589 | 0.32810 |  | -0.00892 | 0.01592 | 0.57530 |
| rs7765781 | C/G | 0.02914 | 0.01095 | 0.00776 |  | 0.02719 | 0.01039 | 0.00883 |  | 0.01650 | 0.01041 | 0.11300 |
| rs7770628 | C/T | -0.01481 | 0.01564 | 0.34340 |  | -0.01753 | 0.01490 | 0.23950 |  | -0.00718 | 0.01493 | 0.63030 |
| rs9457778 | C/T | -0.02121 | 0.01152 | 0.06568 |  | -0.02980 | 0.01097 | 0.00662 |  | -0.02886 | 0.01100 | 0.00871 |

Table S2. MR base single SNP

| SNPs | Congestive Heart Failure | | | | | |  | | Ischemic Stroke | | | | |  | | Atrial Fibrillation | | | | |  | | Arrhythmia | | | | |
| --- | --- | --- | --- | --- | --- | --- | --- | --- | --- | --- | --- | --- | --- | --- | --- | --- | --- | --- | --- | --- | --- | --- | --- | --- | --- | --- | --- |
|  | beta | | se | | *P* value | |  | | beta | | se | | *P* value |  | | beta | se | | | *P* value |  | | beta | se | | *P* value | |
| rs1018234 | 0.40572 | | 0.14821 | | 0.00619 | |  | | -0.01918 | | 0.11281 | | 0.86498 |  | | -0.01900 | 0.20901 | | | 0.92756 |  | | -0.06115 | 0.11096 | | 0.58158 | |
| rs117052562 | 0.11047 | | 0.12761 | | 0.38668 | |  | | 0.16744 | | 0.09684 | | 0.08379 |  | |  |  | | |  |  | | 0.07117 | 0.09689 | | 0.46258 | |
| rs1406888 | 0.09783 | | 0.15143 | | 0.51828 | |  | | 0.23149 | | 0.11510 | | 0.04431 |  | |  |  | | |  |  | | -0.08437 | 0.11336 | | 0.45670 | |
| rs2048327 | -0.00588 | | 0.14366 | | 0.96736 | |  | | -0.22691 | | 0.10927 | | 0.03784 |  | | 0.00000 | 0.19987 | | | 1.00000 |  | | -0.22769 | 0.10762 | | 0.03436 | |
| rs2457574 | -0.01419 | | 0.13837 | | 0.91832 | |  | | -0.22052 | | 0.10524 | | 0.03614 |  | | 0.00000 | 0.19254 | | | 1.00000 |  | | -0.21355 | 0.10365 | | 0.03938 | |
| rs41269133 | -0.04801 | | 0.03934 | | 0.22236 | |  | | 0.01174 | | 0.02988 | | 0.69438 |  | | -0.07589 | 0.04812 | | | 0.11481 |  | | -0.00925 | 0.02954 | | 0.75411 | |
| rs429358 | 0.05691 | | 0.13483 | | 0.67296 | |  | | -0.01723 | | 0.10299 | | 0.86718 |  | | -0.10345 | 0.18621 | | | 0.57851 |  | | 0.07568 | 0.09990 | | 0.44871 | |
| rs520829 | 0.40794 | | 0.14412 | | 0.00465 | |  | | -0.09918 | | 0.10965 | | 0.36574 |  | | -0.24479 | 0.20258 | | | 0.22692 |  | | -0.10968 | 0.10798 | | 0.30974 | |
| rs56393506 | -0.02239 | | 0.03635 | | 0.53785 | |  | | 0.01276 | | 0.02766 | | 0.64471 |  | | -0.04213 | 0.04775 | | | 0.37759 |  | | -0.04038 | 0.02721 | | 0.13782 | |
| rs6415084 | -0.00787 | | 0.03862 | | 0.83851 | |  | | 0.02479 | | 0.02940 | | 0.39909 |  | | -0.04791 | 0.05429 | | | 0.37759 |  | | -0.04058 | 0.02890 | | 0.16026 | |
| rs7770628 | -0.01918 | | 0.03257 | | 0.55586 | |  | | 0.02152 | | 0.02480 | | 0.38547 |  | | -0.04285 | 0.04571 | | | 0.34850 |  | | -0.03028 | 0.02435 | | 0.21378 | |
| rs9457778 | -0.09192 | | 0.17696 | | 0.60346 | |  | | -0.01793 | | 0.13466 | | 0.89408 |  | | -0.61959 | 0.24570 | | | 0.01168 |  | | -0.11288 | 0.13241 | | 0.39393 | |
| All- Inverse variance weighted | -0.00676 | | 0.02267 | | 0.76556 | |  | | 0.01319 | | 0.01642 | | 0.42179 |  | | -0.05873 | 0.02336 | | | 0.01192 |  | | -0.03568 | 0.01284 | | 0.00544 | |
| All- MR Egger | -0.05178 | | 0.02844 | | 0.09866 | |  | | 0.03803 | | 0.02257 | | 0.12297 |  | | -0.03037 | 0.03442 | | | 0.40336 |  | | -0.00947 | 0.01865 | | 0.62268 | |
| SNPs | | Left Ventricular Mass Index | | | | | |  | | Left Ventricular Internal Dimension in Diastole | | | | | | | |  | Left Ventricular Internal Dimension in Systole | | | | | | | |  |
|  |  | beta | | se | | *P* value | |  | | beta | | se | | | *P* value | | |  | beta | | | se | | | *P* value | |  |
| rs1018234 | | -0.06683 | | 0.10346 | | 0.51828 | |  | | -0.11239 | | 0.09852 | | | 0.25396 | | |  | -0.14117 | | | 0.09871 | | | 0.15265 | |  |
| rs117052562 | | -0.03258 | | 0.08808 | | 0.71144 | |  | | -0.01019 | | 0.08344 | | | 0.90276 | | |  | -0.01364 | | | 0.08367 | | | 0.87053 | |  |
| rs1406888 | | 0.04475 | | 0.10415 | | 0.66744 | |  | | 0.04870 | | 0.09915 | | | 0.62328 | | |  | 0.06763 | | | 0.09940 | | | 0.49626 | |  |
| rs2048327 | | 0.17712 | | 0.10012 | | 0.07689 | |  | | 0.10450 | | 0.09536 | | | 0.27316 | | |  | 0.08561 | | | 0.09556 | | | 0.37030 | |  |
| rs2457574 | | 0.16613 | | 0.09645 | | 0.08499 | |  | | 0.10094 | | 0.09178 | | | 0.27138 | | |  | 0.08344 | | | 0.09205 | | | 0.36468 | |  |
| rs41269133 | | -0.05273 | | 0.02434 | | 0.03027 | |  | | -0.05338 | | 0.02317 | | | 0.02125 | | |  | -0.04263 | | | 0.02325 | | | 0.06671 | |  |
| rs429358 | | -0.03202 | | 0.09197 | | 0.72769 | |  | | 0.07392 | | 0.08819 | | | 0.40196 | | |  | 0.13019 | | | 0.08835 | | | 0.14057 | |  |
| rs520829 | | 0.10965 | | 0.10146 | | 0.27983 | |  | | 0.02879 | | 0.09665 | | | 0.76578 | | |  | 0.02227 | | | 0.09682 | | | 0.81810 | |  |
| rs56393506 | | -0.01754 | | 0.02302 | | 0.44603 | |  | | -0.02130 | | 0.02192 | | | 0.33114 | | |  | -0.00865 | | | 0.02197 | | | 0.69364 | |  |
| rs6415084 | | -0.04230 | | 0.02667 | | 0.11269 | |  | | -0.02482 | | 0.02537 | | | 0.32809 | | |  | -0.01424 | | | 0.02542 | | | 0.57536 | |  |
| rs7770628 | | -0.02115 | | 0.02234 | | 0.34367 | |  | | -0.02504 | | 0.02128 | | | 0.23939 | | |  | -0.01026 | | | 0.02132 | | | 0.63039 | |  |
| rs9457778 | | -0.22658 | | 0.12306 | | 0.06560 | |  | | -0.31834 | | 0.11719 | | | 0.00660 | | |  | -0.30830 | | | 0.11751 | | | 0.00870 | |  |
| All- Inverse variance weighted | | -0.02605 | | 0.01320 | | 0.04837 | |  | | -0.02710 | | 0.01235 | | | 0.02828 | | |  | -0.01579 | | | 0.01271 | | | 0.21405 | |  |
| All- MR Egger | | -0.04529 | | 0.01855 | | 0.03476 | |  | | -0.03307 | | 0.01886 | | | 0.10999 | | |  | -0.01700 | | | 0.01957 | | | 0.40560 | |  |


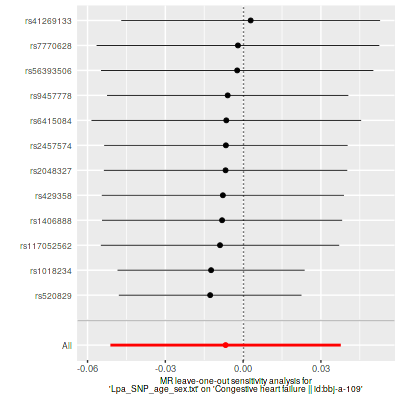


Figure 1-1. MR leave-one-out sensitivity analysis for Lp(a) on Congestive Heart Failure.


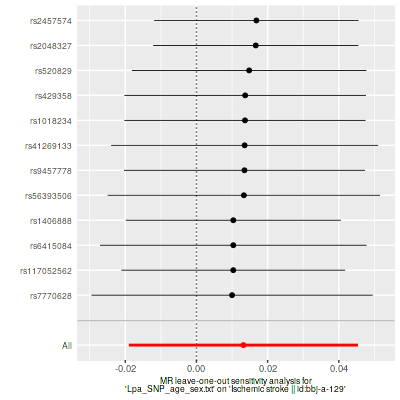


Figure 1-2. MR leave-one-out sensitivity analysis for Lp(a) on Ischemic Stroke


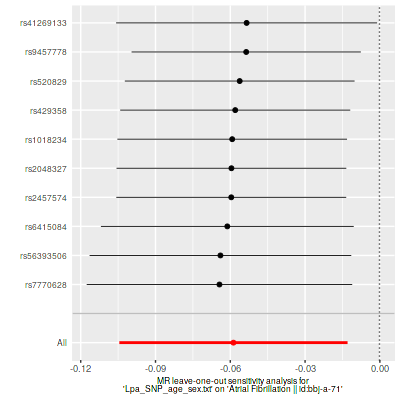


Figure 1-3. MR leave-one-out sensitivity analysis for Lp(a) on Atrial Fibrillation


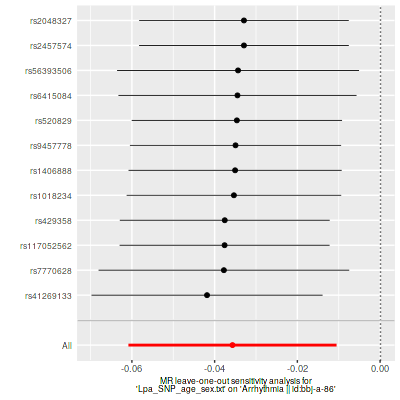


Figure 1-4. MR leave-one-out sensitivity analysis for Lp(a) on Arrhythmia


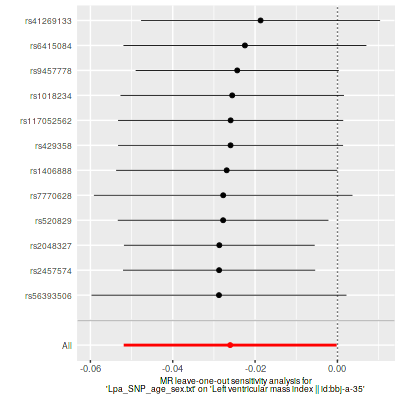


Figure 1-5. MR leave-one-out sensitivity analysis for Lp(a) on Left Ventricular Mass Index


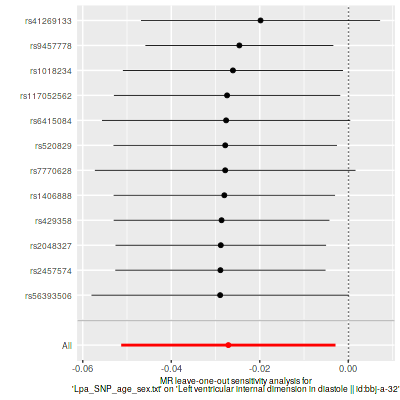


Figure 1-6. MR leave-one-out sensitivity analysis for Lp(a) on Left Ventricular Internal Dimension in Diastole


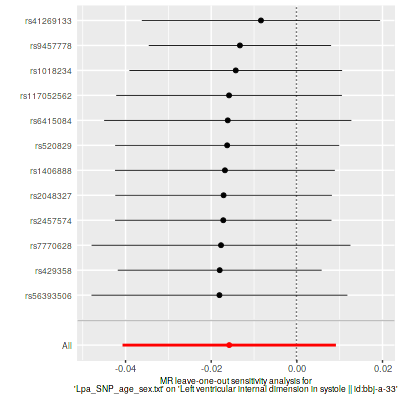


Figure 1-7. MR leave-one-out sensitivity analysis for Lp(a) on Left Ventricular Internal Dimension in Systole


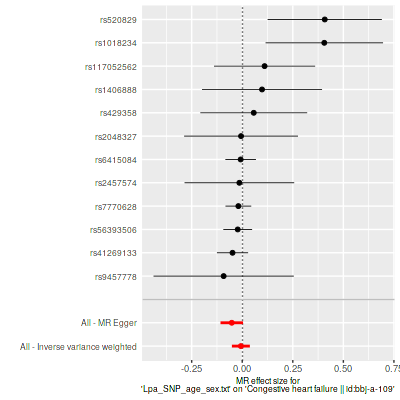


Figure 2-1. Single SNP analysis for Lp(a) on Congestive Heart Failure.


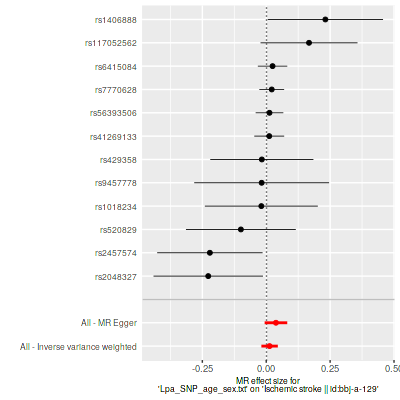


Figure 2-2. Single SNP analysis for Lp(a) on Ischemic Stroke


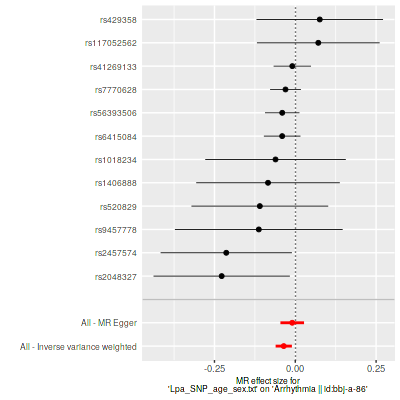


Figure 2-3. Single SNP analysis for Lp(a) on Atrial Fibrillation


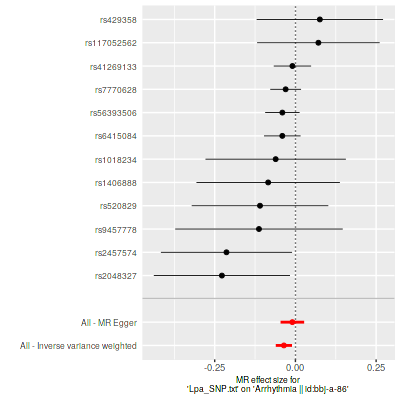


Figure 2-4. Single SNP analysis for Lp(a) on Arrhythmia


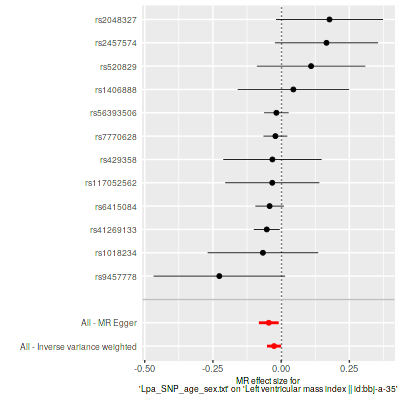


Figure 2-5. Single SNP analysis for Lp(a) on Left Ventricular Mass Index


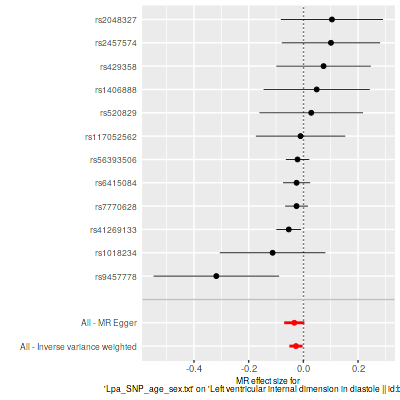


Figure 2-6. Single SNP analysis for Lp(a) on Left Ventricular Internal Dimension in Diastole
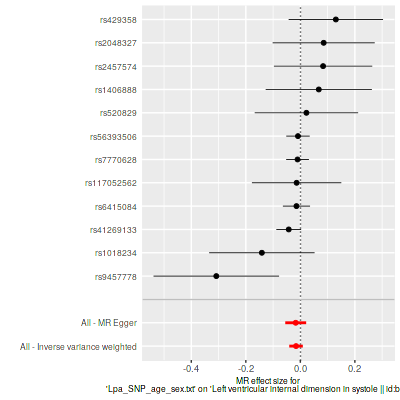


Figure 2-7. Single SNP analysis for Lp(a) on Left Ventricular Internal Dimension in Systole
